# A Cornea Substitute Derived from Fish Scale: 6-Month Followup on Rabbit Model

**DOI:** 10.1155/2014/914542

**Published:** 2014-06-24

**Authors:** Fei Yuan, Liyan Wang, Chien-Chen Lin, Cheng-Hung Chou, Lei Li

**Affiliations:** ^1^Department of Ophthalmology, Zhongshan Hospital of Fudan University, Shanghai, China; ^2^Department of Research and Development, Body Organ Biomedical Corporation, Taipei, Taiwan; ^3^Department of Ophthalmology, Eye & ENT Hospital of Fudan University, Shanghai, China

## Abstract

A fish scale-derived cornea substitute (Biocornea) is proposed as an alternative for human donor corneal tissue. We adopt a regenerative medicine approach to design a primary alternative to the use of fish scale for restoring sight by corneal replacement. Biocornea with corneal multilayer arrangement collagen was implanted to rabbits by pocket implantation. Our study demonstrated the safety and detailed morphologic and physiologic results from the 6 months of followup of rabbit model. In the peripheral Biocornea, the collagen fibrils were arranged in reticular fashion. Slit lamp examination showed that haze and an ulcer were not observed in all groups at 3 months postoperatively while all corneas with Biocornea were clear at both 3 months and 6 months postoperatively. The interface of Biocornea and stromal tissue were filled successfully and without observable immune cells at postoperative day 180. Moreover, the Biocornea was not dissolved and degenerated but remained transparent and showed no apparent fragmentation. Our study demonstrated that the Biocornea derived from fish scale as a good substitute had high biocompatibility and support function after a long-term evaluation. This revealed that the new approach of using Biocornea may yield an ideal artificial cornea substitute for long-term inlay placement.

## 1. Introduction

The human cornea is the transparent outermost surface of the eye and major refractive element of the visual system; its function depends upon its optical clarity. Blindness due to corneal disease results both from numerous degenerative [1], dystrophic [2], infectious, and inflammatory corneal disorders and from corneal damage secondary to ocular surface disease. The epidemiology of corneal blindness is varied and complex, with infectious and nutritional corneal diseases [3], such as trachoma, onchocerciasis (river blindness), and vitamin A deficiency (xerophthalmia), second only to cataract as a cause of blindness worldwide. Although cataracts are responsible in almost half of the patients with vision loss [4], corneal damage and disease are the next largest cause [5]. These disorders are common in developing countries in Asia and Africa and amenable to prevention through public health measures, whereas corneal scarring is the most important cause of reversible blindness in children [6]. Corneal transplantation remains the main method for visual rehabilitation once disease has affected corneal clarity but is dependent on the availability of corneal donor tissue, which is the major limiting factor in developing countries. By contrast, developed countries in the west have more inherited, degenerative, or iatrogenic disorders, such as Fuchs' corneal endothelial dystrophy [7] and postcataract surgery corneal decompensation, which have better prognoses.

Ulceration and trauma are responsible for an additional 1.5 to 2 million new patients with corneal blindness annually [8]. The most successful and widely accepted treatment for corneal blindness worldwide is full-thickness replacement of penetrating keratoplasty (PK). PK was the first method of performing corneal transplantation and remains the most common method. Approximately 80% of all transplants in Australia are still performed this way [9].

Regardless of the technique, the fundamental problem with corneal replacement is a severe shortage of donor tissue, resulting in approximately 10 million [10] untreated patients worldwide. However, the gap between organ demand and donor availability has progressively widened, and the severe shortage of organs for transplantation has resulted in the increasing use of expanded donor criteria, allowing the inclusion of older donors as well as donors with mild disease. Thus, organ donation may involve the risk of the transmittal of unwanted host factors, such as infections and malignancies [11]. Infectious microbes and unexpected diseases that are present in an organ donor have the potential to be transmitted to the transplant recipient. Pretransplant screening costs are high and will escalate as more rigorous testing for an increasing number of transmissible pathogens is implemented [12].

Recently, however, developments in bioengineered corneal substitutes designed to replace the full or partial thickness of damaged or diseased corneas have been reported [13]. These range from fully synthetic prostheses (e.g., keratoprosthesis) made from polymethacrylates, that aim at replacing the cornea's refractive function, to tissue-engineered cell-based constructs [14] and hydrogels [15] that also permit the integration of the implant and regeneration of the host tissues. The most frequent causes of corneal alterations leading to keratoplasty are keratoconus, bacterial infections, poor hygienic contact lens wear, or trauma. Among microbial infections, bacterial infections are the most frequent and are mainly complication. Some side effects of keratoplasty can be infection (keratitis on the new transplanted cornea or endophthalmitis) [16], transplant rejection, vision fluctuation, glaucoma, and bleeding [17], among others which are less reported. Their use is therefore limited to cases in which allogeneic tissue has failed repeatedly or is contraindicated [18]. A number of therapeutic strategies have been adopted to treat donor deficiency. Using human amniotic membrane as a biological substrate is a well-known technique for the management of ocular surface reconstruction in patients with PK [19]. However, there are a few problems with amniotic membrane which still remain unresolved, such as sterile storage for longer periods, the thinness of membrane that affects the suture strength, wrinkling while transplanting, early degradation of the membrane, and the potential danger of the spread of viruses [20] and bacteria. Fish is a good source of collagen. Fish scales are composed of connective tissue protein and collagen (41 to 81%), covered with calcium salts (calcium phosphate and calcium carbonate) [21]. Therefore, fish scale may be an effective alternative source for collagen production [22].

Our goal was to adopt a regenerative medicine approach to design a primary alternative to the use of fish scale for restoring sight by corneal replacement. Specifically, we proposed inducing regeneration of the damaged corneas by implantation of an acellular and decalcified fish scale that serves to facilitate regeneration by emulating the functions of the highly natural extracellular matrix (ECM) scaffolding of the cornea. The substitute is cell-free and of high biocompatibility with host tissue to restore corneal function, thereby avoiding the rejection reaction and the need for long-term steroid use. van Essen et al. [23] demonstrated safety, biocompatibility, and regenerative potential of pocket implantation of a fish scale in rat model. We report here the safety, detailed morphologic, and physiologic results from the 6-month followup of rabbit model. We specifically evaluated the integration and stability of the implanted material and the degree to which the implants enabled regeneration of endogenous epithelium.

## 2. Materials and Methods

### 2.1. Acellular and Decalcified Corneal Scaffold Preparation

The tilapia fish scales were cleaned in distilled water and cellular components were removed using a four-step detergent and enzymatic extraction process as developed by Lin et al. [24]. To increase pore sizes and porosity within the test samples, the acellular tissues were additionally treated with acetic acid. The resulting decellularized fish scales were rinsed extensively and stored. Decalcification of the material was performed by being immersed in 5% nitric acid for 6–16 hours at room temperature (RT). The materials were further decalcified by being immersed in 300 mL of solution A (10% EDTA, 2% nitric acid) for 2-3 days at 4°C with renewal of solution A daily depending on the degree of mineralization of the scales. After decalcification, samples were rinsed with 70% ethanol and stored in sterilized PBS at 4°C for study. Finally, acellular and decalcified fish scale-derived cornea substitute (8.0 ± 0.8 mm [diameter], 250 ± 50 *μ*m [thickness]), consisting of collagen type I, was used for implantation.

### 2.2. Scanning Electron Microscopy

Scanning electron microscopy was used to examine the surface morphology of acellular corneal scaffold. The acellular scaffolds were fixed in 2.5% glutaraldehyde in PBS (pH 7.4) for 10 min. The fixative was then aspirated. After being washed in PBS, scaffolds were dehydrated in a graded series of ethanol solutions. After critical point drying (Quorum Technologies, model E3100, Guelph, Ontario, Canada), the samples were sputtered with gold using a SEM coating system (SPI, Sputter Coater11430, West Chester, PA, USA), and the probes were examined by scanning electron microscopy (JEOL, JSM-5610, Tokyo, Japan).

### 2.3. Histopathological Evaluation

After euthanizing the rabbits with carbon dioxide, the isolated corneas were fixed in 4% paraformaldehyde in 0.1 M phosphate buffer (pH 7.4) for 2 hours at room temperature or overnight at 4°C. After gradient dehydration in 10%, 15%, and 20% sucrose in 0.1 M phosphate-buffered saline (PBS) each for 4 hours, the cornea was cut into 2 halves and embedded at optimal cutting temperature compound (composed of glacial acetic acid, ethyl alcohol, and buffered formalin). Sections were cut on a microtome (Leica RM2165; Leica Microsystems GmbH, Wetzlar, Germany) at 5 um and stained with hematoxylin and eosin (HE) for histological examination. All eyes were analyzed for the organization of the epithelium, stroma, and endothelium and observed for infiltration of immune cells and ingrowth of corneal cells into the corneal scaffold. The immune infiltrate was characterized based on morphology.

### 2.4. Surgical Technique

Sixteen New Zealand White rabbits, aged 1-2 months and weighing 2–2.5 kg, were used in the study. Animals were anaesthetized using an intramuscular injection of ketamine hydrochloride (35 mg/kg; Parnell Laboratories, Alexandria, Australia) and xylazine hydrochloride (5 mg/kg; Troy Laboratories, Smithfield, Australia). One eye of each rabbit was chosen at random for surgery. The contralateral eye of the implanted rabbit served as the control. Two drops of tetracaine (Chauvin Pharmaceuticals, Kingston upon Thames, UK) were applied to the selected eye. Following anesthesia, the rabbit eye to be implanted was prolapsed anteriorly to improve surgical access. An anterior lamellar dissection was performed using a crescent blade. The centre of the cornea was first marked with a keratoplasty trephine (Solan; Xomed Surgical Products Inc., Jacksonville, FL, USA). A guarded diamond knife (Storz, Bausch and Lomb, USA) was set to 75% corneal depth and a 3 mm circumferential incision was made to enable the formation of a pocket incision and hence preservation of the corneal nerves. A depth of 75% has been previously shown to be the optimal position for an implant for nutritional flow (glucose and oxygen) in cornea stromal. A deep lamellar dissection was then made, down to the posterior third of the cornea. A closed dissection technique was used by maintaining the anterior lamellar flap in its original position close to the stromal bed and the crescent knife (Sharpoint, Angiotech, Vancouver, Canada) was sandwiched between the 2 layers. With forceps gently holding the anterior flap in place, the knife was gradually advanced perpendicular to the cornea curvature in a gentle sweeping motion, thus splitting the cornea along a single lamellar plane. Sandwiching the blade between the stromal layers (and ensuring no tissue distortion occurs) resulted in an even dissection, increasing the likelihood of the dissection staying in the same tissue plane throughout. A 4 mm diameter implant was then folded and inserted into the pocket. Pocket incisions have been previously shown to maintain the nervous innervation of the cornea compared to a conventional LASIK like lamellar flap. The implantation was done sequentially starting with implant. Following insertion, the implant was unfolded using a Paton spatula (Storz Instrument, Bausch & Lomb, USA) with a gentle sweeping motion. The 3 mm incision was then sutured with interrupted 10/0 nylon sutures. All rabbits received the following topical medications after implantation: neomycin-polymyxin B-dexamethasone ointment (Maxitrol; Alcon Laboratory, Inc., Fort Worth, TX, USA) four times per day, Pred Forte (Allergan; Irvine, California, USA) four times per day, atropine 1% (Allergan; Irvine, California, USA) twice daily, and a lubricating viscous gel (Vidisic Gel; Mann Pharma, Berlin, Germany) once a day for 1 week. Sutures were removed selectively in all rabbits at either 2 or 3 weeks after implantation.

### 2.5. Diagnosis of Corneal Recovery

External examinations of each eye were done initially once daily for first 2 weeks and then on a once-a-week basis over the entire course of the study. Detailed slit-lamp examinations of each rabbit were performed (in a double-masked manner) every other week. Eyes were examined for the presence of corneal perforation, vascularization, or infection. The diagnosis of corneal recovery was based on a subjective evaluation of corneal curvature and stromal stability, including the presence of epithelium migration by slit-lamp examination and fluorescent staining.

## 3. Results and Discussion

**Figure 1:**
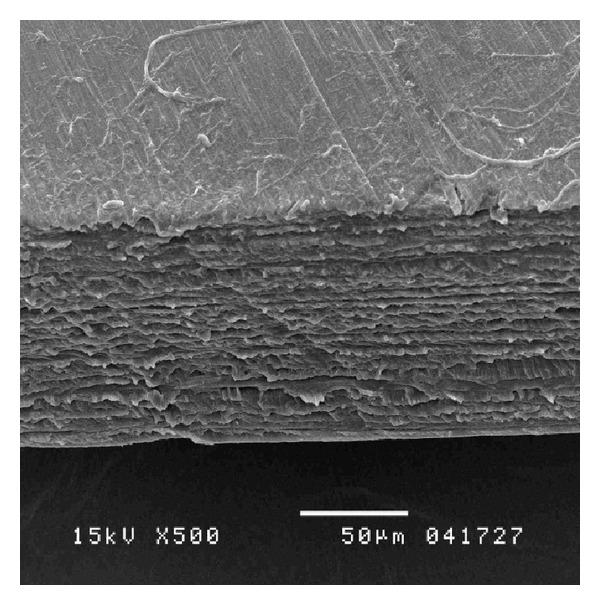
The multilayer arrangement of collagen within Biocornea.

Comparing to the native cornea, the 3D arrangement of the collagen similar to the corneal was studied. In the central Biocornea, the cutting edge consisted of multilayer collagen fibrils (Figure 1). The collagen layer was orthogonally arranged and was formed by parallel bundles of fibrils, whereas the multilayer was a continuous plate of parallel fibers. The fibrils of the neighboring 2 layers crossed each other at an angle of about 90°. In the peripheral Biocornea, the collagen fibrils were arranged in reticular fashion. The mechanical behavior of IOP resistant is directly affected by the arrangement of the fibers in its layer. Most pressurized organs and organisms are reinforced by a crossed-helical fiber array [25], where fibers are wrapped in left- and right-handed helices around the long axis of the structure [26]. Crossed-helical fiber reinforcement permits constant pressure and volume of eyeball easily [27] and resisting occasional burst of IOP. These structures can stick easily because collagen fibers are oriented parallel to the direction of the vertical force [28]. Moreover, collagen multilayer structure also can maintain water content to ca 84%.

The helical arrangement of fibers makes sense for structures that are flexible [29]. But cornea needs to remain in shape during daily scratch of eyelid, resisting external forces that could change the smoothness of surface and prevent the incision. For the first 3 days after the injury, obvious conjunctival edema and eyelid swelling were observed for each rabbit. By day 7, most of the eyes were covered with the regenerating of pocket wound but small epithelial defects were detected. From day 10 on, the average areas of wound defects were significantly healing.

Of the 16 rabbits that underwent surgery, 24 rabbit eyes received Biocornea and 8 rabbit eyes as control group. There were four sham control eyes from two rabbits, where the surgery was performed without implantation. One of the rabbits with Biocornea and two rabbits as control developed microbial keratitis within the first week of implantation and were excluded from the study. A total of 11 rabbits had Biocornea and 3 rabbits as control had successfully proceeded. One rabbit in the Biocornea group developed progressive vascularization starting from 4 to 6 weeks and hence was terminated earlier than originally planned. The remaining 10 rabbits with Biocornea were maintained to the original predetermined time points without any problems. They also all revealed no immune response over the 6-month period of study.

**Figure 2:**
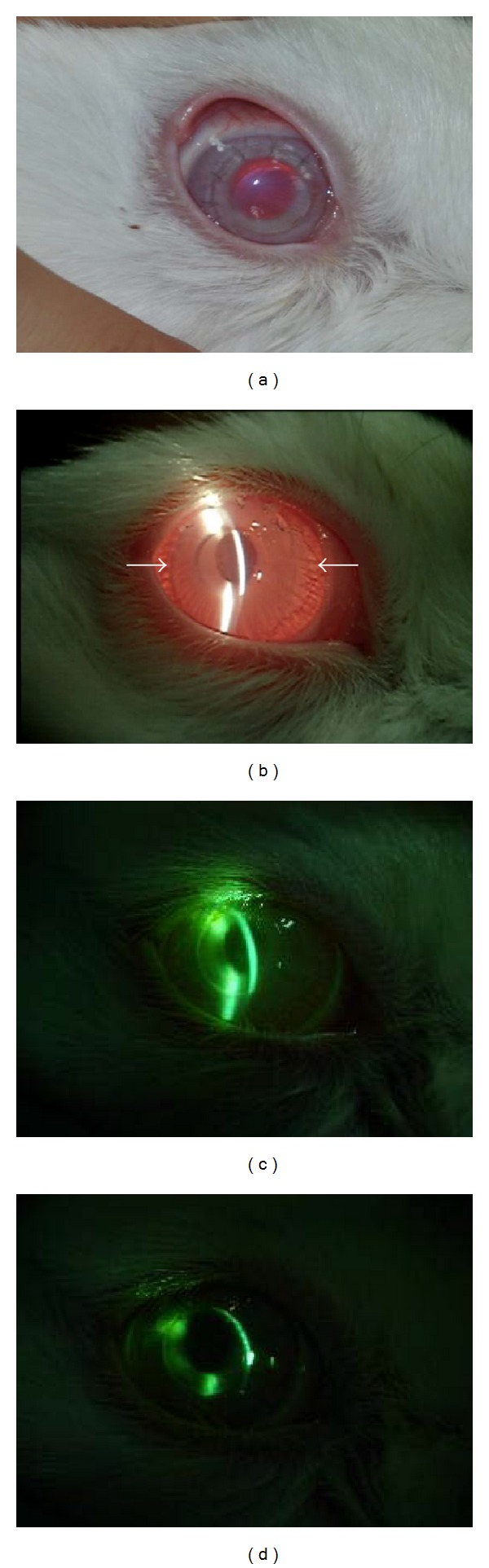
Five sutures were proceeded on the entrance of pocket with 10-0 silk and node buried inside; arrow indicated the transparency implant (a). Slit-lamp examination. Arrows indicate a successful inlay implant, which is almost imperceptible because of its extreme thinness after one week (b). Representative slit-lamp micrographs of the rabbit corneas. Cornea with Biocornea at 3 months (c). Cornea with Biocornea at 6 months (d).

**Figure 3:**
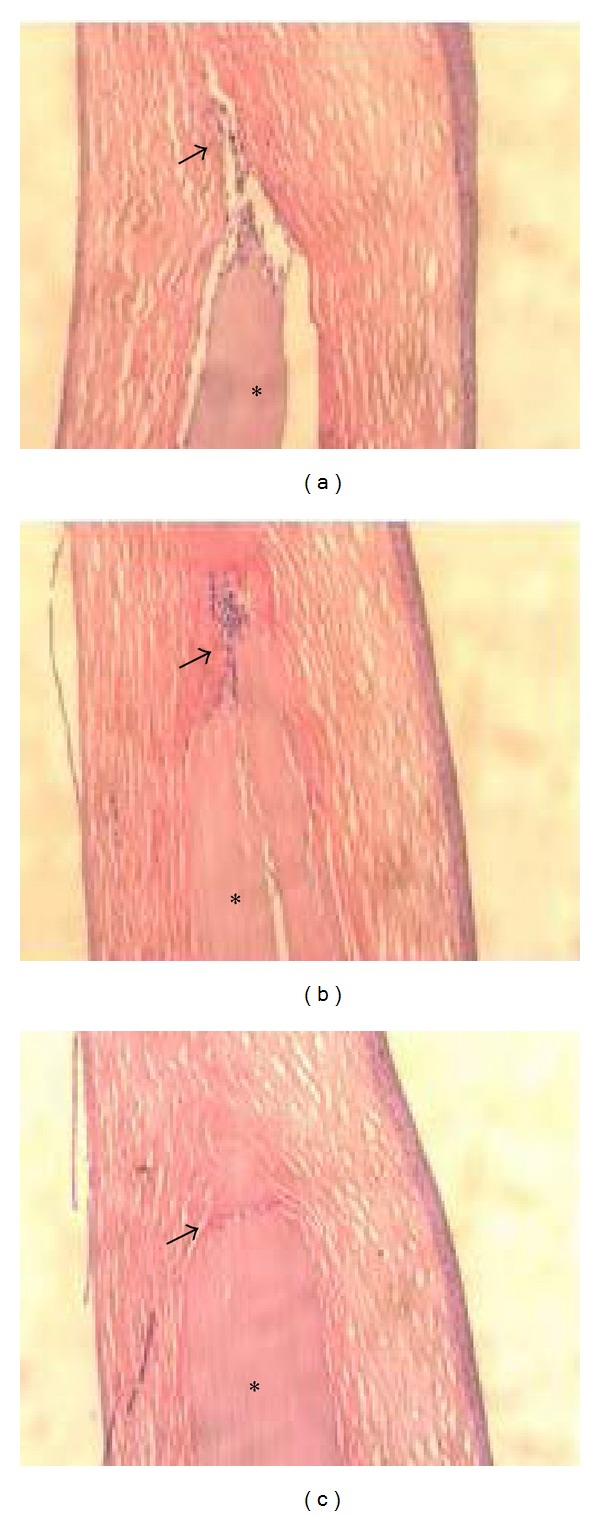
Histology revealed the interface of Biocornea and stromal tissue. Asterisk indicates the location of Biocornea and arrow indicated the immune cells. After 1 week implant, some immune cells infiltrate around the Biocornea and exhibit some space in between tissue and implant (a). There was significant decrease of immune cells and tight junction of implant and tissue after 3 months (b). The implant revealed high biocompatibility from disappearance of immune cells and integration of tissue after 6 months (c).

Slit-lamp examination showed that all control and sham-operated corneas were haze-free at both 3 and 6 months postoperatively (Figures 2(b) and 2(c)). Haze and an ulcer were not observed in all groups at 3 months postoperatively (Figure 2(c)) while all corneas with Biocornea were clear at both 3 months and 6 months postoperatively. The eyes were enucleated at first week, 3 months, and 6 months postoperatively. In the Biocornea group, a gross examination of eye specimens showed that the interface of Biocornea could not integrate perfectly with the stroma and infiltrate some immune cells (Figure 3(a)) after 1 week. Biocornea remained transparent and well-rounded (Figure 2(a)). The interface of Biocornea and stromal tissue were filled much better at 3 months postoperatively. Also, the transparency of Biocornea was still very clear, and few immune cells infiltrates seem to have obviously changed (Figure 3(b)). However, the integration was filled successfully and without observable immune cells at postoperative day 180 (Figure 3(c)). Moreover, Biocornea was not dissolved and degenerated but remained transparent and showed no apparent fragmentation.

*In vivo*, biocompatibility of Biocornea depends on various factors including the material itself (permeable or nonpermeable polymer), the surgical procedure, the test animals, and the postoperative conditions. Of these factors, the surgical procedure plays an important role in determining the tolerance of an inlay. In most mammals including humans, the bundles enter the cornea at the periphery in a radial fashion parallel [30] to the corneal surface exerting important trophic influences on the corneal epithelium and contributing to the maintenance of a healthy ocular surface.

## 4. Conclusions

Our study demonstrated that the Biocornea derived from fish scale as a good substitute had high biocompatibility and support function after a long-term (180 days) evaluation. This revealed that the new approach of using Biocornea may yield an ideal artificial cornea substitute for long-term inlay placement. This new approach greatly decreased the biodegradation and improved its stability performance as a cornea inlay.
